# Evaluating Brightness and Spectral Properties of Click Beetle and Firefly Luciferases Using Luciferin Analogues: Identification of Preferred Pairings of Luciferase and Substrate for *In Vivo* Bioluminescence Imaging

**DOI:** 10.1007/s11307-020-01523-7

**Published:** 2020-09-14

**Authors:** Giorgia Zambito, Natasa Gaspar, Yanto Ridwan, Mary P. Hall, Ce Shi, Thomas A. Kirkland, Lance P. Encell, Clemens Löwik, Laura Mezzanotte

**Affiliations:** 1 Erasmus Medical Center, Radiology and Nuclear Medicine, Rotterdam, The Netherlands; 2 Erasmus Medical Center, Molecular Genetics, Rotterdam, The Netherlands; 3 Medres medical research GmBH, Cologne, Germany; 4 Percuros B.V, Leiden, The Netherlands; 5 Promega Corporation, Madison, WI USA; 6 Promega Biosciences Incorporated, San Luis Obispo, CA USA; 7 CHUV Department of Oncology, University of Lausanne, Lausanne, Switzerland

**Keywords:** Bioluminescence, *In vivo* imaging, Luciferase, Emission spectrum, Luciferin

## Abstract

**Purpose:**

Currently, a variety of red and green beetle luciferase variants are available for bioluminescence imaging (BLI). In addition, new luciferin analogues providing longer wavelength luminescence have been developed that show promise for improved deep tissue imaging. However, a detailed assessment of these analogues (*e.g*., Akalumine-HCl, CycLuc1, and amino naphthyl luciferin (NH_2_-NpLH2)) combined with state of the art luciferases has not been performed. The aim of this study was to evaluate for the first time the *in vivo* brightness and spectral characteristics of firefly (Luc2), click beetle green (CBG99), click beetle red 2 (CBR2), and Akaluc luciferases when paired with different d-luciferin (d-LH2) analogues *in vivo*.

**Procedures:**

Transduced human embryonic kidney (HEK 293T) cells expressing individual luciferases were analyzed both *in vitro* and in mice (*via* subcutaneous injection). Following introduction of the luciferins to cells or animals, the resulting bioluminescence signal and photon emission spectrum were acquired using a sensitive charge-coupled device (CCD) camera equipped with a series of band pass filters and spectral unmixing software.

**Results:**

Our *in vivo* analysis resulted in four primary findings: (1) the best substrate for Luc2, CBG99, and CBR2 in terms of signal strength was d-luciferin; (2) the spectra for Luc2 and CBR2 were shifted to a longer wavelength when Akalumine-HCl was the substrate; (3) CBR2 gave the brightest signal with the near-infrared substrate, NH_2_-NpLH2; and (4) Akaluc was brighter when paired with either CycLuc1 or Akalumine-HCl when paired with d-LH2.

**Conclusion:**

We believe that the experimental results described here should provide valuable guidance to end users for choosing the correct luciferin/luciferase pairs for a variety of BLI applications.

**Electronic supplementary material:**

The online version of this article (10.1007/s11307-020-01523-7) contains supplementary material, which is available to authorized users.

## Introduction

Bioluminescence imaging (BLI) is a well-known, non-invasive technique employed during preclinical studies to track cells and monitor biological processes in living animals [1–3]. BLI is performed by capturing the light generated by a luciferase upon exogenous substrate (*e.g.*, d-luciferin (d-LH2)) addition to report real-time, cellular, and molecular events [4].

Over the last decade, the bioluminescence toolbox has greatly expanded [1, 5, 6]. Novel luciferin analogues have been introduced that enhance light emission *in vivo* and increase detection sensitivity in deeper tissues [7]. Cycluc1 has been shown to enhance emission of codon optimized firefly luciferase (Luc2), especially in the brain. Furthermore, this system provides slightly red-shifted emission resulting in deeper light penetration and less scattering of the bioluminescence signal [8, 9]. Likewise, Akalumine-HCl has a spectral peak in the near infrared (NIR) (677 nm) and enhanced emission with Luc2 when administered at low concentration [10]. Akalumine-HCl paired with the recently engineered Akaluc luciferase is even brighter, although the spectral peak is blue-shifted to 650 nm [11]. Amino naphthyl luciferin (NH_2_-NpLH2) represents another new substrate with potential for deeper tissue BLI [12]. This substrate was shown to emit in the NIR with a peak of 740 nm when reacting with an engineered version of click-beetle luciferase (CBR2). CBR2 can also utilize d-LH2 and this combination was shown to improve imaging in black fur mice compared with Luc2/ d-LH2.

Research into the development of improved BLI reagents has generally focused on bioluminescence systems comprised of compatible luciferase/luciferin pairings [13–18]. Most comparative studies have been performed using d-LH2. For example, Miloud et al. compared firefly (Luc2) and click beetle luciferases *in vivo* with d-LH2 as substrate and concluded that click beetle green (CBG99) has sensitivity and total photon yield comparable with click beetle red [15]. In other studies, Luc2 was shown to have improved performance compared with a red-shifted firefly mutant (PpyRE9) and CBG99 for brain imaging [16, 17], but d-LH2 was the only substrate examined. A direct comparison (either *in vitro* or *in vivo*) of emission spectra and relative brightness of bioluminescence systems comprised of different luciferase enzymes in combination with novel luciferins has, to date, not been reported.

Here, we provide a detailed *in vitro* and *in vivo* analysis of brightness and emission spectra for four luciferases when combined with four different substrates using a CCD camera equipped with a series of band pass filters and spectral unmixing software. We anticipate that the results of this comparative analysis will help enable researchers to choose the best enzyme/substrate pairs for different BLI applications. In addition, our findings revealed that depending on the luciferase/luciferin pair, a wide range of spectral emission peaks (*i.e.*, multicolored luciferases) is available that could broaden the BLI toolbox in the future for multiplex analysis both *in vitro* and *in vivo.*

## Materials and Methods

### Animals

Animal experiments were approved by the Bioethics Committee of Erasmus MC, Rotterdam, The Netherlands, and performed in accordance with national guidelines and regulations established by the Dutch Experiments on Animal Act (WoD) and by the European Directive on the Protection of Animals used for scientific purpose (2010/63/EU). BALB/C nude (females) were obtained from Charles River Laboratory (The Netherlands). All mice aged 6–8 weeks were provided access to food and water *ad libitum* and were hosted in the animal facility at the Erasmus MC, Rotterdam, The Netherlands.

### Cell Line

Human embryonic kidney cells (HEK 293T) were cultured in Dulbecco’s Modified Eagle’s Medium (DMEM) (Sigma, St. Louis, MO, USA) supplemented with 10 % of FBS and 1 % Penicillin-Streptomycin. The culture was incubated at 37 °C with 5 % CO_2_.

### Lentivirus Production

Virus production and cell transduction were performed under appropriate biosafety level conditions (ML-II) in accordance with the National Biosafety Guidelines and Regulations for Research on Genetically Modified Organisms. Procedures and protocols were reviewed and approved by the EMC Biosafety Committee (GMO permit 99-163). The lentiviral plasmids pCDH-EF1-CBG99-T2A-copGFP, pCDH-EF1-Luc2-T2A-copGFP, and pCDH-EF1-CBR2-T2A-copGFP were previously described [12, 15]. The plasmid pCDH-EF1-Akaluc-T2A-copGFP was produced by inserting the sequence of Akaluc (amplified with specific primers from pcDNA3 Venus-Akaluc plasmid from RIKEN BRC repository) without stop codon using BamHI and NotI sites in pCDH-EF1-MCS-T2A-copGFP vector. Lentiviruses were produced by transfection of HEK 293T packaging cells with three packaging plasmids (pCMV-VSVG, pMDLg-RRE, pRSV-REV; Addgene, Cambridge, MA, USA) and the lentiviral vector plasmids as previously described in details [16].The supernatant containing lentiviral particles were collected after 48 and 72 h. Subsequent quantification of the virus was performed using a standard antigen-capture HIV p24 ELISA (ZeptoMetrix Corporation, NY, USA).

### Cell Transduction and Transfection

Cell transduction was performed by culturing HEK 293T cells in DMEM supplemented with 10 % of FBS and 1 % of Penicillin-Streptomycin at the density of 200,000 cells in a T25-flask with 5 ml of medium. Expression in the lentiviral plasmid is driven by housekeeping elongation factor 1α (EF1) promoter. Cells were transduced with MOI 1 of either pCDH-EF1-Luc2-T2A-copGFP, pCDH-EF1-CBG99-T2A-copGFP, pCDH-EF1-CBR2-T2A-copGFP, or pCDH-EF1-Akaluc-T2A-copGFP lentivirus plus with polybrine (hexametride bromide, Sigma-Aldrich) at the final concentration of 8 μg/ml. Transgene expression was confirmed by the presence of the super bright green fluorescent protein copGFP from the copepod *Potentilla plumata* (excitation/emission maximum = 482/502 nm).

### Flow Cytometry to Sort Stable Cell Lines

Positive stable clones were sorted for comparable levels of copGFP expression by cell sorting (BD-FACS ARIA III, BD Biosciences). Forward and side scatters were also drawn to eliminate cellular debris from the analysis and to select highly positive cells for GFP.

### *In Vitro* BLI

Transduced cells were plated at a density of 2 × 10^4^ cells per well in a black 96-well plate (Greiner Cell Star®) and imaged in 100 μl of D-PBS. Bioluminescence signal from wells was measured with IVIS® spectrum system (PerkinElmer, Boston, MA, USA) every 5 min after substrate addition (final concentration of each substrate was 0.1 mM). All *in vitro* measurements were acquired after 1 min at 37 °C using a 30-s acquisition time with an open filter or using a series of band pass filters ranging from 520 to 800 nm. Data were analyzed by the Living Image software version 4.3 (PerkinElmer). Data in every well were normalized for fluorescence emission detected using a GloMax®-Multi plate reader.

### *In Vivo* BLI

Each stable expressing cell line was injected subcutaneously 1 × 10^5^ cells/50 μl. The number of animals was chosen according to power analysis (*p* value at least < 0.05 and power 95 %) considering that we expected from the data generated *in vitro* that the brightest BL system would differ by 1–2 orders of magnitude *in vivo*. Mice (*N* = 3 per group) received two different cell lines, one in each flank. Animals were then imaged after intraperitoneal injection of d-LH2 substrate (150 mg/kg), NH_2_-NpLH2 substrate (220 mg/kg), CycLuc1 (7.6 mg/kg), and Akalumine-HCl substrate (50 mg/kg). These doses were chosen based on maximum solubility (for CycLuc1 and Akalumine-HCl), tolerability in mice, and maximum attainable signal based on previous findings. Mice were randomly assigned and anesthetized by isoflurane inhalation prior to performing BLI imaging. The person performing the subcutaneous injections was blind as to the cells being injected. Images were acquired with the IVIS® spectrum small animal imager system (PerkinElmer). Light was measured using open filter and a series of 20 nm wavelength band filters from 520 to 800 nm with acquisition time of 30 s during a time of about 30 min after substrate injection (kinetic analysis). Emission signals were measured with the Living Image software® version 4.3 (Perkin Elmer).

### Statistical Analysis

All statistical analyses were performed using the GraphPad Prism 6 software and one-way ANOVA followed by Tukey’s post-test. *p* values < 0.05 were considered statistically significant.

## Results

### *In Vitro* Evaluation of Emission Properties for Different Combinations of Luciferase Variant and Luciferin Analogue

The aim of this study was to evaluate *in vitro* and *in vivo* light emission and spectral differences between four luciferases (Luc2, CBG99, CBR2, and Akaluc) when combined with d-LH2 or three luciferin analogues (NH_2_-NpLH2, Akalumine-HCl, or CycLuc1) for bioluminescence imaging (BLI). To compare the different emissions, HEK 293T cells stably expressing each of the four luciferases were treated with substrates (0.1 mM) and imaged at 37 °C. Equimolar expression of each luciferase was achieved by selecting cells for GFP emission.

**Fig. 1.**
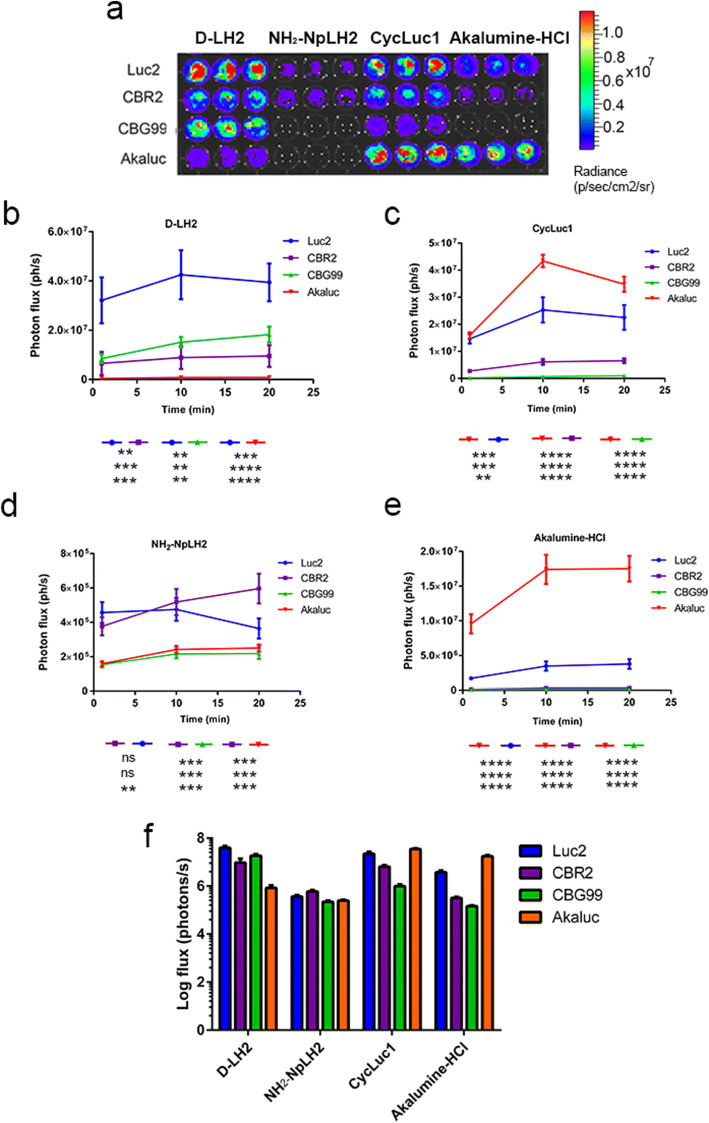
(**a**) Bioluminescence profiles for Luc2, CBR2, CBG99, and Akaluc luciferases combined with four different luciferin analogues in live cells. (**b**-**f**) Photon flux (ph/s) in HEK 293T cells expressing individual luciferases upon addition of substrates (0.1 mM) was quantified using an exposure time of 30 s. Statistical analysis (*N* = 3) was performed using one-way ANOVA followed by Tukey’s *T* test (*p < 0.01 for Luc2/d-LH2 compared with all combinations with the exception of Akaluc/Cycluc1 which was not significantly different).

We found that the luciferase/luciferin pairs yielding the highest photon emission (*p* value < 0.001) were Luc2/d-LH2 and Akaluc/CycLuc1 when the substrate was added at a concentration of 0.1 mM. The combinations of Luc2/CycLuc1, Akaluc/Akalumine-HCl, CBG99/d-LH2, and CBR2/d-LH2 produced ~ 2-fold fewer photons (Fig. 1a), while cells expressing CBG99 were much less efficient (~ 100-fold dimmer with NH_2_-NpLH2/Akalumine-HCl; 10-fold dimmer with CycLuc1) (Fig. 1a). CBR2-expressing cells were more promiscuous compared with CBG99 cells. However, they generated 10-fold less luminescence (compared with Luc2/d-LH2) with Akalumine-HCl and NH_2_-NpLH2. The CBR2-expressing cells gave a signal comparable with Luc2/d-LH2 with CycLuc1. Finally, Akaluc produced similar luminescence intensity when either Akalumine-HCl or CycLuc1 was used as substrate (Fig. 1a). Akaluc also showed nearly 100-fold lower signal with d-LH2 or NH_2_-NpLH2 compared with Akalumine-HCl and CycLuc1.

### *In Vivo* Emission Spectrum of Luciferases Detected Using a Series of 20 nm Band Pass Filters

The day of injection, HEK 293T cells (expressing the various luciferases) were prepared at a concentration of 2 × 10^6^ cells/ml in PBS and fluorescence emission measured at IVIS, confirming the comparable average expression of GFP (Supplementary Fig. 1). Following subcutaneous injection of 1 × 10^5^ HEK 293T cells (expressing the various luciferases) into both flanks of mice, images were acquired after injection of d-LH2 (150 mg/kg), NH_2_-NpLH2 (220 mg/kg), Akalumine-HCl (50 mg/kg), or CycLuc1 (7.6 mg/kg). We used the optimal concentration for each given substrate based on previous literature [8, 10, 12, 19]. For d-LH2, this was 150 mg/kg [19]. Because of poor aqueous solubility, CycLuc1 and Akalumine-HCl were injected at 7.6 mg/kg (5 mM in saline) and 50 mg/kg (33 mM in saline), respectively [8, 10]. We previously demonstrated that the solubility of NH_2_-NpLH2 allows injection of a maximal dose of 220 mg/kg (60 mM in saline) and that it produces significantly higher photon fluxes than a dose of 150 mg/kg [12]. Multiple acquisitions using a series of 20 nm band pass filters were performed with an exposure time of 30 s. The BLI measurements were performed at the time of peak of emission after injection of the luciferins into sedated animals.

**Fig. 2.**
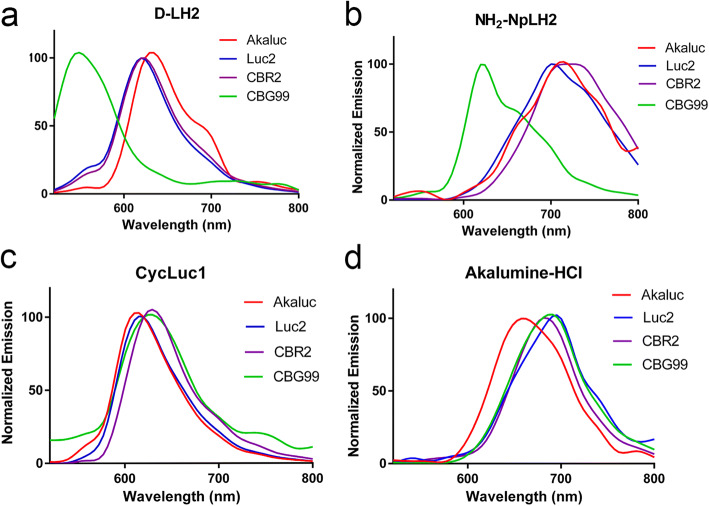
*In vivo* (BALB/C) emission spectra for different combinations of luciferase (Luc2, CBG99, CBR2, or Akaluc; expressed in HEK393T cells implanted subcutaneously in the flanks) and luciferin or luciferin analogue. (**a**) D-LH2 (150 mg/kg), (**b)** NH_2_-NpLH2 (220 mg/kg), (**c**) CycLuc1 (7.6 mg/kg), and (**d)** Akalumine-HCl (50 mg/kg); substrates were injected intraperitoneally). Spectral data was acquired 15–20 min after injection.

In terms of emission spectra, Luc2/d-LH2, CBG99/d-LH2, CBR2/d-LH2, and Akaluc/d-LH2 produced peaks at 610 nm, 540 nm, 620 nm, and 640 nm, respectively (Fig. 2a). NH_2_-NpLH2 caused a red shift of the peak of emission with all the luciferases (Luc2, 700 nm; Akaluc, 720 nm; CBR, 730 nm; and CBG99, 620 nm) (Fig. 2b). In contrast, when CycLuc1 was used as a substrate, the emission peak for each luciferase was in the range of 620 nm (Luc2 and Akaluc were green shifted towards 600 nm and CBG99 and CBR2 were red-shifted towards 640 nm) (Fig. 2c). Akaluc/Akalumine-HCl, also referred to as the AkaBLI system [11], produced a peak of emission at 660 nm while the other luciferases peaked in the NIR (~ 680 nm) when paired with Akalumine-HCl (Fig. 2d).

### *In Vivo* Comparison of Brightness of Luciferase/Luciferin Pairing

**Fig. 3.**
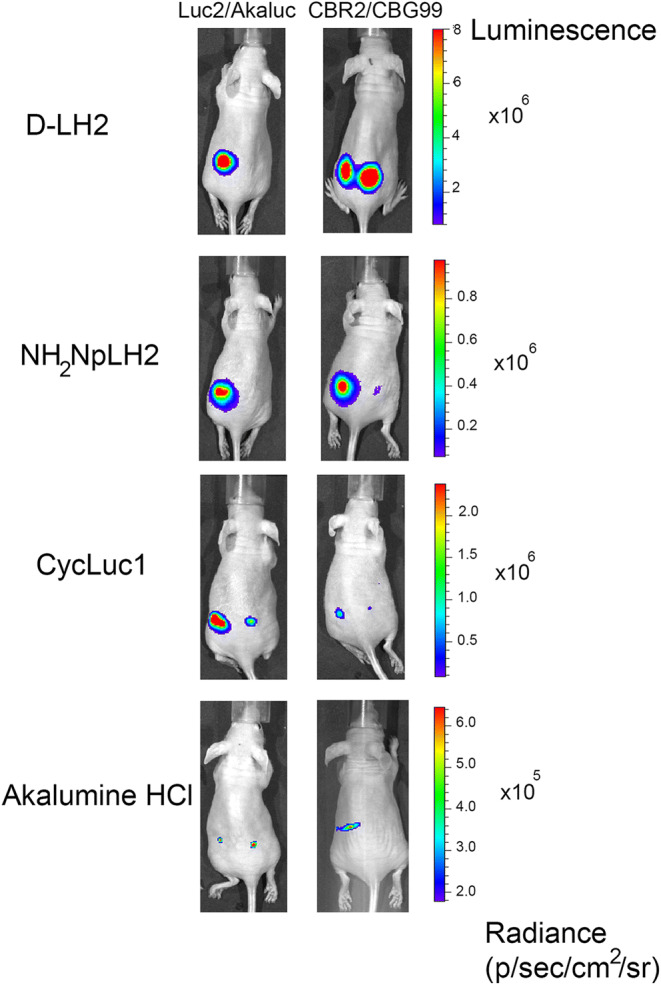
Superficial bioluminescence imaging of BALB/C mice in which 1 × 10^5^ HEK293T cells transduced with Luc2 and Akaluc or CBR2 and CBG99 were implanted subcutaneously into the left and right flanks of mice, respectively, and treated (intraperitoneally) with (**a) **D-LH2 (150 mg/kg), (**b)** NH_2_-NpLH2 (220 mg/kg), (**c)** CycLuc1 (7.6 mg/Kg), and (**d)** Akalumine-HCl (50 mg/kg). Imaging data was collected using open filters and with an exposure time of 30 s. Average luminescence is reported as photons/s/cm^2^/sr.

**Fig. 4.**
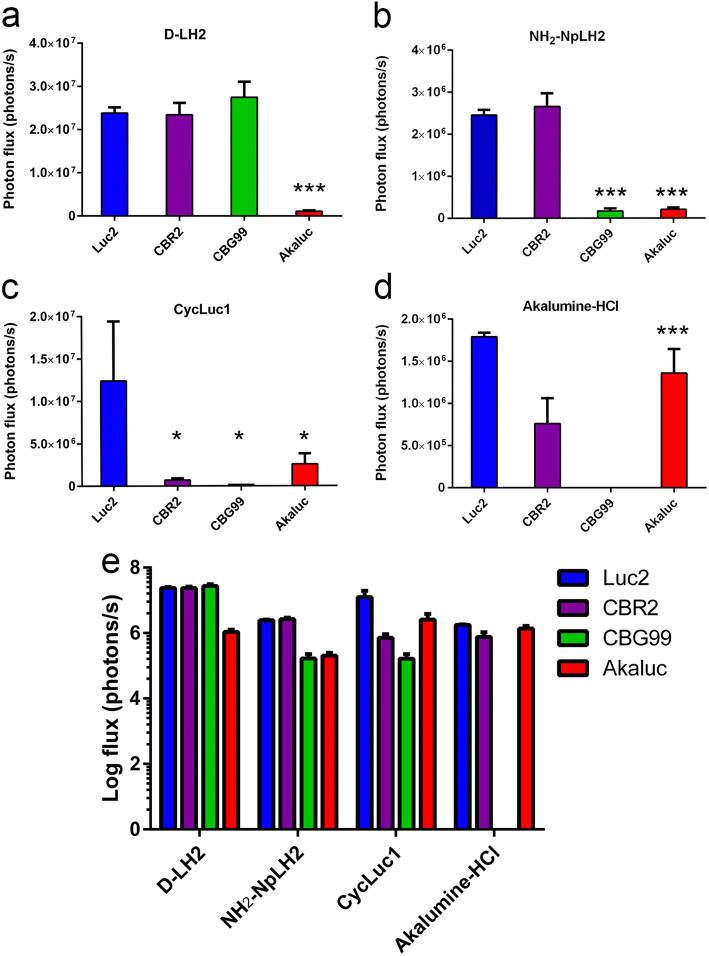
Quantification of photon flux (ph/s) measured *in vivo* for all combinations of luciferase and substrate (d-LH2 (**a**), Akalumine-HCl (**b**), CycLuc1 (**c**), and NH_2_-NpLH2 (**d**). Combined data is also presented in logarithmic scale (**e**). Statistical analysis of data was performed using one-way ANOVA followed by Tukey’s post-test (**p < 0.0019; ***p < 0.001; ****p < 0.0001).

Next, we compared the total emission of each luciferase *in vivo* with d-LH2 or the luciferin analogues. Figure 3 shows the representative bioluminescent images of nude mice where CBG99, Luc2, CBR2, and Aka-Luc-expressing cells were implanted, and each of the different substrates was injected intraperitoneally. The data in Fig. 4 represents signals at peak of emission which differs slightly between BLI systems (Supplementary Fig. 2). Luc2, CBG99, and CBR2 paired with d-LH2 produced the highest signals which were 20-fold higher compared with Akaluc/d-LH2 (*p* value < 0.001) (Fig. 4a). When NH_2_-NpLH2 was used as a substrate, Luc2 and CBR2 produced approximately 10-fold higher signal output (*p* value, 0.001) compared with both CBG99 and Akaluc (Fig. 4b). When CycLuc1 was used as a substrate, the strongest signal was detected for Luc2/Cycluc1. Akaluc, CBR2, and CBG99 paired with Cycluc1 produced ~ 5, 16, and 70-fold lower signal output, respectively (Fig. 4c). When Akalumine-HCl was used as a substrate, Luc2 and Akaluc produced ~ 2-fold higher signal compared with CBR2, and there was no detectable signal for CBG99 (Fig. 4d).

The luciferase/luciferin pairs that gave the highest photon yields *in vivo* were Luc2/d-LH2, Luc2/CycLuc1, CBG99/d-LH2, and CBR2/d-LH2 (1–2 × 10^7^ ph/s). The following luciferin/luciferase pairs produced approximately 10-fold fewer photons: Akaluc/Cycluc1, Akaluc/Akalumine-HCl, Luc2/Akalumine-HCl, Luc2/NH_2_-NpLH2, and CBR2/NH2-NpLH2. Finally, the following pairs produced nearly 100-fold fewer photons: CBR2/CycLuc1, CBR2/Akalumine-HCl, CBG99/Cycluc1, CBG99/NH_2_-NpLH2, and Akaluc/NH_2_-NpLH2 (Fig. 4e, Table 1).

**Table 1:**
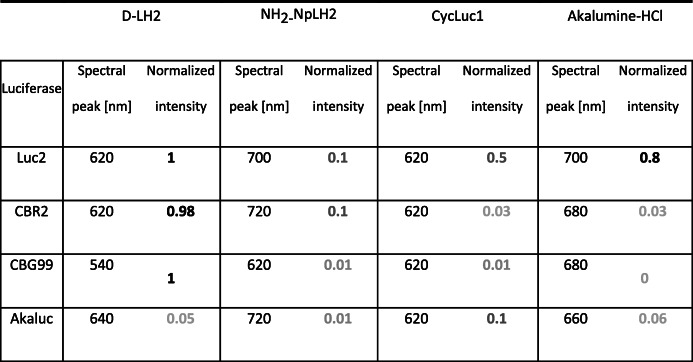
*In vivo* emission intensity relative to Luc2/D-LH2

## Discussion

A variety of new luciferase enzymes and novel substrate analogues emerging in recent years have resulted in better tools for *in vivo* BLI. One example is CBR2/NH_2_-NpLH2, which was engineered specifically for enhanced NIR emission to improve imaging resolution in deeper tissues [12]. Another example is Akaluc/Akalumine-HCl [11], an engineered pair offering improved *in vivo* sensitivity. Another relatively new substrate, Cycluc1, has shown *in vivo* utility (including more efficient crossing of the blood brain barrier compared with d-LH2) when used in combination with the already well-established Luc2 [8]. With the emergence of these and other new bioluminescence systems, we felt it would be of interest and potential benefit for the *in vivo* BLI community, particularly for those interested in dual color readouts, to analyze different pairings of luciferase/substrate using a common set of test parameters. Here, we report on the photon yields and spectral characterization of Luc2, CBG99, CBR2, and Akaluc luciferases combined with four different substrates (d-LH2, NH_2_-NpLH2, Cycluc1, and Akalumine-HCl) both *in vitro* and *in vivo*. Our goal was to use these parameters to compare the various luciferase/substrate combinations in a standard subcutaneous *in vivo* BLI model, with the intention to provide guidance for the *in vivo* BLI community when choosing appropriate systems for specific applications involving dual color detection. Note the longer emission wavelengths for CBR2/NH_2_-NpLH2 and Akaluc/Akalumine-HCl provide a sensitivity advantage in deeper tissue [11, 12] that will not be fully realized in a subcutaneous model. However, we postulated that the peak emissions in the NIR for these systems would provide excellent spectral separation from shorter wavelength signals nonetheless.

We have demonstrated *in vitro* that at a relatively low, but biologically relevant (*in vivo*) substrate concentration (0.1 mM), three of the four luciferases give maximum signal when combined with d-LH2. The exception was Akaluc, which produced more photons when using either Cycluc1 or Akalumine-HCl as substrate. We observed the same trend in a low-depth, superficial *in vivo* tissue model. Though we did not examine deeper tissues in this study, we predict based on our results that the red-shifted NIR systems (CBR2/NH_2_-NpLH2, Akaluc/Akalumine-HCl, and Luc2/Akalumine-HCl) would perform best.

To evaluate spectral properties *in vivo* as a way to determine the potential for multiplexing, we used the same superficial, subcutaneous model where different luciferase expressing cell lines were injected into the backs of mice. This minimally invasive model allowed us to determine the light emission characteristics for different BLI systems using a small cohort of animals. Based on the analysis, we are able to recommend new combinations of luciferases with distinct colors having potential for multiplexing with a single substrate in superficial tissue *e.g.*, CBG99/d-LH2 (540 nm) and CBR2/d-LH2 (620 nm) (examples of spectral unmixing showed in Supplementary Fig. 3); CBG99/d-LH2 (540 nm) and Luc2/d-LH2 (610 nm); and Luc2/Akalumine (680 nm) and Akaluc/Akalumine (650 nm). Such an approach could be useful for analyzing multiple parameters or biological processes in animals using either engrafted cells or transgenes expressed in particular tissues or organs, and as part of a single imaging session requiring fewer animals.

Successful multiplexing of luminescence systems with different emission spectra relies on the acquisition of images using multiple filters followed by accurate, algorithm-based spectral unmixing to resolve the contributions from each luciferase to total light output. This can be a challenge with shorter wavelength systems (*e.g.*, CBG99/d-LH2), as they tend to shift their apparent emission peak to significantly longer wavelengths when imaged in deeper tissues or even in superficial tissue when using mice with dark fur [20–22]. For these more challenging imaging targets, it is therefore desirable to use bioluminescence pairs that emit in the NIR (> 650 nm), as emission peaks are essentially constant in this range of the spectrum [22, 23]. In this regard, we found that click beetle luciferases have high photon emission with NH_2_-NpLH2 [12] and that there is a broad spectral separation between CBG99 (620 nm) and CBR2 (720 nm) (spectral unmixing is shown in Supplementary Fig. 2). However, before giving serious consideration to this pair with NH_2_-NpLH2 as a multiplexing opportunity for deep tissue imaging in mice, it will likely be necessary to improve the photon yield for CBG99/NH_2_-NpLH2.

## Electronic Supplementary Material

Supplementary Figure 1 a) Representative fluorescent image of the cells solutions before injection. b) Quantification of the average fluorescent signals from 0.5 ml solution containing 2x10^6^ cells/ml. Experiment was done in triplicate. (PDF 188 kb)

Supplementary Figure 2 *In vivo* kinetics of D-LH2, NH_2_-NpLH2, CycLuc1 and Akalumine-HCl at various time points ranged between 5 and 30 minutes after injection of substrates. Data are presented as means (n=3) and SD and curves are generated using lowess smoothing function. (PNG 85 kb)

High resolution image (TIFF 82 kb)

Supplementary Figure 3 Representative spectral unmixing images of CBR2 and CBG99 luciferases. Mice were imaged after administration of NH_2_-NpLH2 (**a**) or of D-LH2 (**b**) substrates using a series of band pass filters at IVIS spectrum (Perkin Elmer). A spectral unmixing algorithm applied to the images extracted and measured each luciferase contribution and generated the two reported spectra of emission. (PNG 104 kb)

High resolution image (TIFF 134 kb)
