# Supplementary figures and images for: Evaluating Brightness and Spectral Properties of Click Beetle and Firefly Luciferases Using Luciferin Analogues: Identification of Preferred Pairings of Luciferase and Substrate for In Vivo Bioluminescence Imaging
Source: Mol Imaging Biol. 2020 Sep 14;22(6):1523–31. doi: 10.1007/s11307-020-01523-7 (PMC7666294; doi:10.1007/s11307-020-01523-7)

**a**

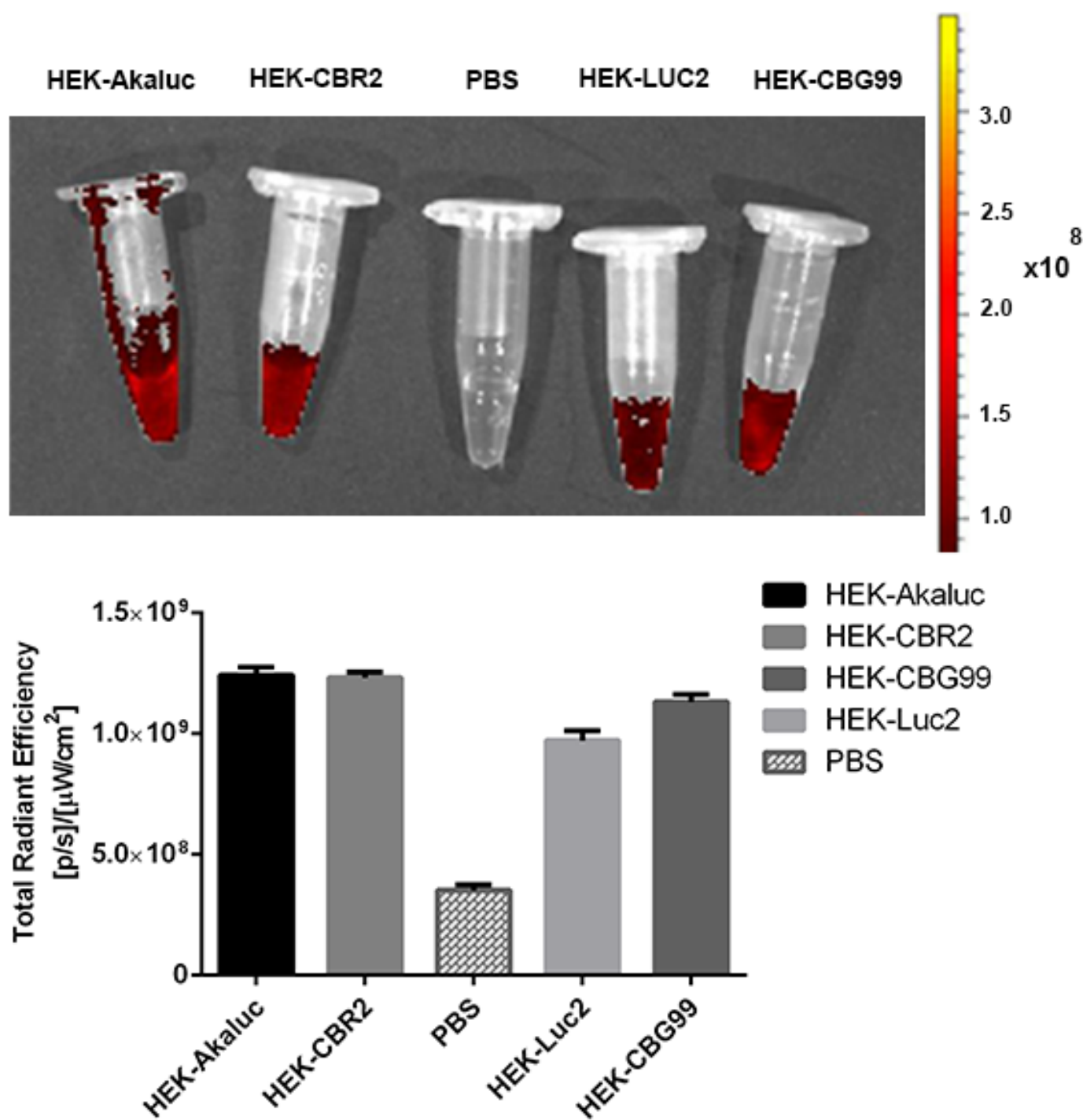

Supplement: Supplementary file 1 — a) Representative fluorescent image of the cells solutions before injection. b) Quantification of the average fluorescent signals from 0.5 ml solution containing 2x10^6^ cells/ml. Experiment was done in triplicate. (PDF 188 kb) [file 11307_2020_1523_MOESM1_ESM.pdf]

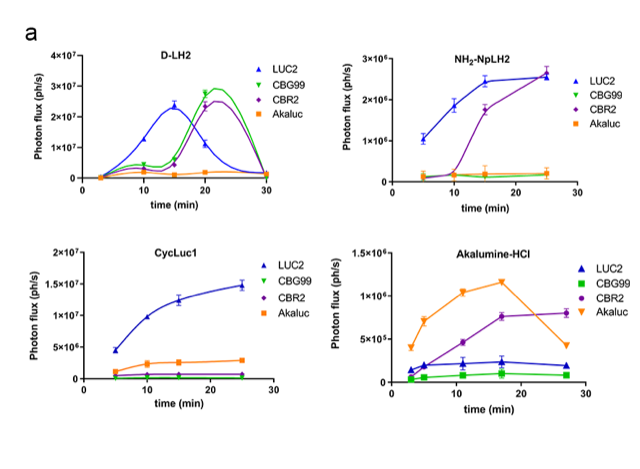

Supplement: Supplementary file 2 — In vivo kinetics of D-LH2, NH2-NpLH2, CycLuc1 and Akalumine-HCl at various time points ranged between 5 and 30 minutes after injection of substrates. Data are presented as means (n=3) and SD and curves are generated using lowess smoothing function. (PNG 85 kb) [file 11307_2020_1523_Fig5_ESM.png]

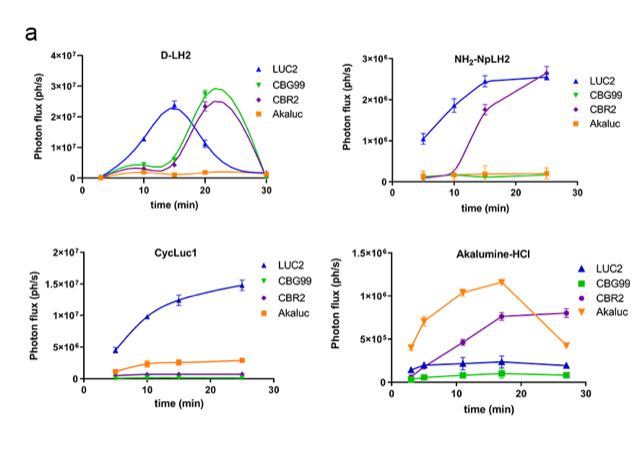

Supplement: Supplementary file 3 — High resolution image (TIFF 82 kb) [file 11307_2020_1523_MOESM2_ESM.tiff]

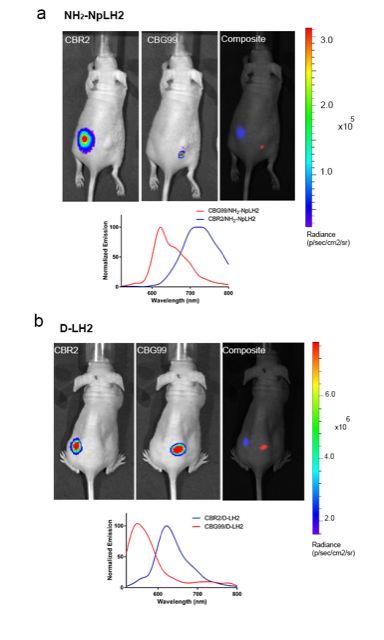

Supplement: Supplementary file 4 — Representative spectral unmixing images of CBR2 and CBG99 luciferases. Mice were imaged after administration of NH2-NpLH2 (a) or of D-LH2 (b) substrates using a series of band pass filters at IVIS spectrum (Perkin Elmer). A spectral unmixing algorithm applied to the images extracted and measured each luciferase contribution and generated the two reported spectra of emission. (PNG 104 kb) [file 11307_2020_1523_Fig6_ESM.png]

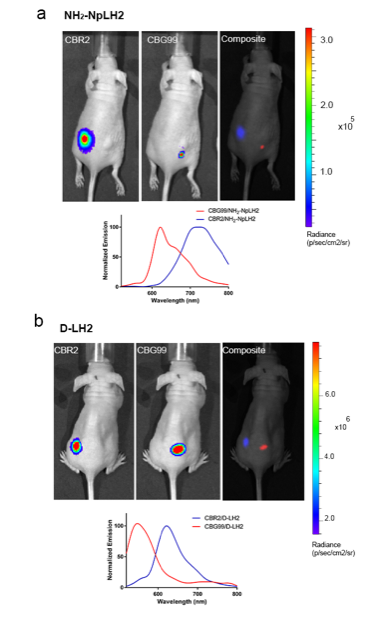

Supplement: Supplementary file 5 — High resolution image (TIFF 134 kb) [file 11307_2020_1523_MOESM3_ESM.tiff]
